# Supplementary material for: MetaRibo-Seq measures translation in microbiomes
Source: Nat Commun. 2020 Jun 29;11:3268. doi: 10.1038/s41467-020-17081-z (PMC7324362; doi:10.1038/s41467-020-17081-z)
Supplement: Supplementary file 10 — Supplementary Data 7 [file 41467_2020_17081_MOESM10_ESM.zip › File2/Confidence_High_Taxonomy/358250_out.krona.html]

Javascript must be enabled to view this page.

members
magnitude
magnitudeUnassigned
count
unassigned
taxon
rank

358250\_out

26

superkingdom
26
2

26
976
phylum

class
26
200643

order
171549
26

family
815
26

26
816
genus


SRS013951\_contig\_number\_18259SRS014855\_contig\_number\_7703SRS015694\_contig\_number\_contig-100\_7742.87026SRS015854\_contig\_number\_13438SRS016897\_contig\_number\_contig-100\_1504.1505SRS016954\_contig\_number\_contig-100\_305.96312SRS017247\_contig\_number\_contig-100\_22.69188SRS017433\_contig\_number\_17446SRS019693\_contig\_number\_9563SRS023346\_contig\_number\_6554SRS024075\_contig\_number\_13920SRS024625\_contig\_number\_contig-100\_659.88579SRS045004\_contig\_number\_2331SRS045645\_contig\_number\_15768SRS057717\_contig\_number\_2224SRS062654\_contig\_number\_15781SRS063813\_contig\_number\_4888SRS098644\_contig\_number\_36497SRS101376\_contig\_number\_contig-100\_3693.107927SRS104084\_contig\_number\_contig-100\_4046.4047SRS1055034\_contig\_number\_13289SRS1055067\_contig\_number\_contig-100\_1473.103942SRS140645\_contig\_number\_1224SRS142980\_contig\_number\_contig-100\_73.52076SRS144362\_contig\_number\_45227SRS893327\_contig\_number\_4108
species
28116
26
